# Friend or Foe: The Relativity of (Anti)oxidative Agents and Pathways

**DOI:** 10.3390/ijms23095188

**Published:** 2022-05-06

**Authors:** András Szarka, Tamás Lőrincz, Péter Hajdinák

**Affiliations:** 1 Laboratory of Biochemistry and Molecular Biology, Department of Applied Biotechnology and Food Science, Budapest University of Technology and Economics, Szent Gellért tér 4, H-1111 Budapest, Hungary; lorincz.tamas@vbk.bme.hu (T.L.); hajdinak.peter@vbk.bme.hu (P.H.); 2 Biotechnology Model Laboratory, Faculty of Chemical Technology and Biotechnology, Budapest University of Technology and Economics, Szent Gellért tér 4, H-1111 Budapest, Hungary

**Keywords:** iron, reactive oxygen species, oxidative stress, ascorbate, cell death, cancer

## Abstract

An element, iron, a process, the generation of reactive oxygen species (ROS), and a molecule, ascorbate, were chosen in our study to show their dual functions and their role in cell fate decision. Iron is a critical component of numerous proteins involved in metabolism and detoxification. On the other hand, excessive amounts of free iron in the presence of oxygen can promote the production of potentially toxic ROS. They can result in persistent oxidative stress, which in turn can lead to damage and cell death. At the same time, ROS—at strictly regulated levels—are essential to maintaining the redox homeostasis, and they are engaged in many cellular signaling pathways, so their total elimination is not expedient. Ascorbate establishes a special link between ROS generation/elimination and cell death. At low concentrations, it behaves as an excellent antioxidant and has an important role in ROS elimination. However, at high concentrations, in the presence of transition metals such as iron, it drives the generation of ROS. In the term of the dual function of these molecules and oxidative stress, ascorbate/ROS-driven cell deaths are not necessarily harmful processes—they can be live-savers too.

## 1. Introduction

Sometimes it is hard to decide whether a molecule is a drug or a poison. Similarly, it is hard to state whether a process is beneficial or harmful. Three entities were chosen to show their relative role in cell fate decision.

As aerobic life is dependent on oxygen, the processes involving its transport, storage, and (bio)chemical reactions involve the fourth most abundant metal on earth: iron. Iron is an obligate nutrient for almost all cells. This element is a critical component of numerous proteins involved in energy production, biosynthetic, metabolic, and detoxification processes, oxygen transfer and storage or host defence. The most common outcome of systemic iron deficiency is anaemia: reduced oxygen-carrying capacity of the blood caused by a decrease in mature erythrocytes leading to tissue hypoxia. On the other hand, excessive amounts of free iron in the presence of oxygen can promote the production of potentially toxic reactive oxygen species (ROS). Although oxygen is essential for aerobic life, ROS are formed as natural by-products during normal cell activity [1]. They can result in persistent oxidative stress, which in turn can lead to cellular, tissue, and organ damage, and eventually to cell death and organ failure. At the same time, ROS—at strictly regulated levels—are essential to maintaining the redox homeostasis, and they are engaged in many cellular signalling pathways, so their total elimination is not expedient. Vitamin C or ascorbic acid establishes a special link between ROS generation/elimination and cell death. At low concentrations, it behaves as an excellent antioxidant and has an important role in ROS elimination, and in this way gives excellent protection against ROS-driven cell death. However, at high concentrations, especially in the presence of transition metals such as iron, it drives the generation of hydrogen peroxide and hydroxyl radicals that lead to ROS-induced cell death. Considering the elevated sensitivity of cancer cells to ROS and oxidative stress, this can represent a potential therapeutic approach in the fight against cancer. Considering the dual function of these molecules and oxidative stress, vitamin C/ROS-driven cell deaths are not necessarily harmful processes—they can be live-savers too.

In the forthcoming chapters, insights are given into the dual function and the interlacement of these three entities.

## 2. Iron, an Element with Multiple Roles

Iron deficiency is accompanied by the reduced oxygen-carrying capacity of the blood caused by a decrease in mature erythrocytes leading to tissue hypoxia. Similarly, genetic defects in the haemoglobin beta chain—as seen in *β*-thalassemia—result in microcytic anaemia, and excessive compensatory absorption of iron is associated with iron overload. This form of dysregulated iron level is also present in hereditary haemochromatoses, which result from defects in the elements of the iron homeostatic pathway (described below). Excessive amounts of free iron in the presence of oxygen can promote the production of potentially toxic ROS through the Haber–Weiss (Equation (1)) and Fenton-type (Equation (2)) reactions. Iron overload and iron-mediated ROS production can result in persistent oxidative stress, which in turn can lead to cellular, tissue, and organ damage, and eventually to organ failure; thus, the cellular level of its redox-active form is under tight regulation by iron chaperons, iron carrier proteins, and transport mechanisms.

O_2_·^−^ + H_2_O_2_ → O_2_ + HO· + OH^−^ (1)

Fe^2+^ + H_2_O_2_ → Fe^3+^ + HO· + OH^−^ (2)

### 2.1. Absorption, Transport, and Cellular Uptake of Iron

Iron homeostasis is controlled by its absorption, as no excretion pathways are known. The absorption of heme-bound iron is preferred through an unknown mechanism—possibly involving receptor-mediated endocytosis, while uptake of non-heme Fe^2+^ is achieved by enterocytic divalent metal-ion transporter 1A-I (DMT1) with a H^+^ cotransport in the proximal duodenum [2,3]. Due to the presence of oxygen, iron is predominantly present in the poorly soluble Fe^3+^ form and needs to be reduced for absorption. This is achieved by the brush-border membrane ascorbate-dependent transmembrane ferrireductase duodenal cytochrome b (Dcytb) on the apical side of enterocytes or non-enzymatically [4,5]. Both the transport and the reduction process are promoted by the presence of an acidic environment provided by the Na^+^/H^+^ exchanger-3 (NHE3) [5].

Iron inside enterocytes can either be stored in the ferritin protein complex when iron demand is low or exported through the basolateral membrane by the efflux pump ferroportin 1 (FPN1). FPN1 is present in the membrane of cells important in the recycling of iron (enterocytes, hepatocytes, and reticuloendothelial macrophages), and its level is negatively controlled by the iron-regulatory peptide hormone hepcidin produced mainly by hepatocytes [5]. Overall, 95% of plasma iron is bound to the glycoprotein transferrin (Tf) in the Fe^3+^ form, while the remaining 5% is non-transferrin bound iron (NTBI). Fe^2+^ is exported by FPN1, and its oxidation is catalysed by the multicopper ferroxidase proteins hephaestin—present both as a membrane-anchored extracellular protein and in the cytosol of enterocytes—and ceruloplasmin—present at the extracellular surface of hepatocytes and astrocytes and excreted into the plasma [6]. All cells of the body need iron; however half of the total amount of iron stored (~2–2.5 g) is present in circulating erythrocytes in the form of haemoglobin. As with the senescence of erythrocytes, the recycling of heme-derived iron through erythrophagocytosis by the reticuloendothelial system (mainly red pulp macrophages and hepatic Kupffer cells) is of great importance to iron homeostasis [5]. Low serum levels of Tf-bound iron can lead to anaemia and the hindrance of iron-dependent biochemical processes, while its elevation may result from infections, inflammation, cancer, or liver disorders.

### 2.2. Cellular Iron Homeostasis: Regulation and Dysregulation

**Figure 1:**
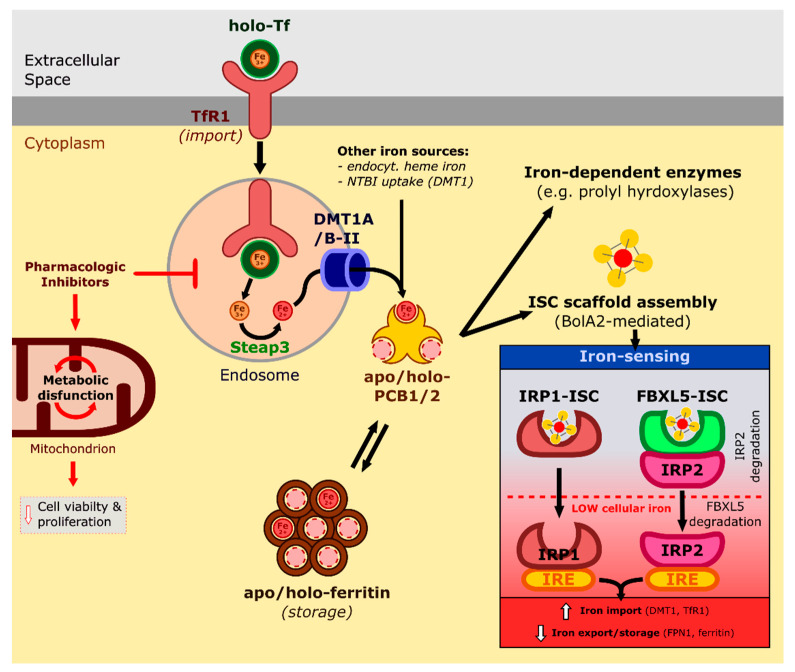
Transport and regulation of intracellular iron levels. Transferrin (Tf)-bound iron is internalised through the transferrin receptor (TfR1) pathway. Inside the endosome, imported ferric iron is released and reduced to ferrous iron by Steap3. DMT1A/B-II transports ferrous iron to the cytoplasm, where it binds to PCB1/2. Cytoplasmic iron can either be stored in ferritin or incorporated into iron-dependent enzymes or the assembly of iron–sulfur cluster (ISC) scaffolds. Low levels of intracellular iron can hinder ISC assembly, thus activating the IRP1/2 iron-sensing pathways as the activity of both IRP1 and FBXL5-IRP2 is dependent on ISC availability. Activated IRP1/2 can bind to iron response elements (IREs) which regulate iron import, export and storage.

Cytosolic iron is essential for the proliferating cell, as numerous processes involve iron-containing proteins, while proteins with iron–sulfur clusters (ISC) are involved in energy metabolism and signalling (Figure 1). Iron chaperons and iron-containing complexes mask the reactivity of redox-active iron; hence, the possibility of undesirable side reactions with molecular oxygen can be limited.

Most cell types import iron through the receptor-mediated endocytosis of transferrin receptor (TfR1)-bound holo-Tf (Figure 1) [5]. Inside the acidified endosome, Tf releases Fe^3+^, and while apo-Tf-TfR1 is recycled, six-transmembrane epithelial antigen of the prostate 3 (Steap3) reduces iron to Fe^2+^, which is exported to the cytosol by DMT1A/B-II [3]. A recent study showed that pharmacologic inhibition of lysosomal activity in cancer cells induces metabolic dysfunction in mitochondria and decreases cell viability and proliferation, which can be successfully alleviated by iron supplementation. These results point towards iron homeostasis as the critical metabolic process in physiologically functioning lysosomes in cancer cells [7].

Iron originating from either the endocytosed TfR1 endosome, the endocytosed heme phagolysosome, or non-transferrin bound iron imported through DMT1 is taken up in the cytoplasm by the iron chaperons poly r(C) binding protein 1 and 2 (PCB1 and 2) (Figure 1). Both PCB1 and 2 can transfer iron directly to ferritin or iron-dependent enzymes such as prolyl hydroxylase [8]. It was shown recently that PCB1 takes part directly in the cytosolic assembly of ISC scaffolds, as it forms a complex with GSH-bound Fe and BolA-like protein 2 (BolA2), for which glutaredoxin 3 (Glrx3) functions as a chaperone [9].

Intracellular iron level is regulated by the ISC containing iron sensor proteins IRP1 and 2, which can bind key regulatory elements—iron response elements (IREs)—to target protein mRNAs (Figure 1). Iron scarcity activates the iron-starvation response through IRP1/2, which facilitates iron import and represses export and storage by regulating protein levels within the iron import (DMT1, TfR1) and export/storage (FPN1/ferritin) pathways. IRP1, also called aconitase (ACO1), is a metabolic enzyme that during insufficient iron levels fails to incorporate ISCs and can thus bind to IREs. IRP2 activity is dependent on FBXL5 binding, which serves as a ubiquitination signal for IRP2 proteasomal degradation. FBXL5 contains an iron-sensing ISC domain, which stabilises its interaction with IRP2 when sufficient cellular iron is present; iron scarcity, however, leads to FBXL5 ubiquitination and hence IRP2 activation. Recently, it was shown that IRP2 binding affinity to IREs is also influenced by ISC synthesis suppression—independent from the IRE1 pathway—and tissue oxygen level. At the same time, the IRP2-mediated elevation of the cellular iron pool can sensitise the cells towards iron overload-dependent cell death [10].

The intracellular iron level is also sensed by the hypoxia-inducible factor (HIF) pathway, as the hydroxylation of the proline residues in HIF α is catalysed by the oxygen and iron-dependent prolyl-hydroxylases. Activated HIFs consequently influence cellular iron homeostasis through interaction with IRPs or proteins within the import/storage/export pathways through hypoxia-response elements (HREs) [11]. During cellular iron overload, the master orchestrator of antioxidant response NRF2 can act to decrease the level of unbound iron by increasing ferritin and FTN1 expression to counteract the increased oxidative threat [12].

### 2.3. Ferritinophagy and Iron Overload-Induced Toxicity

It was demonstrated that the pathomechanism of various conditions is associated with ferritinophagy-induced labile iron overload, increased oxidative stress and lipid peroxidation. To replenish cellular iron pools, besides modulating import and export, sequestered iron is also liberated from ferritin by the process of ferritinophagy, a specialised form of autophagy, dependent on the lysosomal cargo receptor nuclear receptor coactivator 4 (NCOA4) [13]. Ferritinophagy plays a crucial role during physiological processes which demand large amounts of iron, as demonstrated during red cell development in cellular, zebrafish [14], and murine [15] models. Inhibition of PCB1 or NCOA4 results in reduced heme synthesis and hemoglobin formation and perturbation of erythroid regulatory systems.

Excess ferritinophagy, however, can contribute to cellular iron overload, which renders the cell prone to oxidative stress while consequent oxidative damage sensitises the cells towards the novel type of regulated cell death: ferroptosis [16]. While ferroptosis inducers were originally developed to function as genotype-selective antitumor agents [17,18,19], ever-growing evidence suggests their implications in iron overload-dependent processes such as cardiovascular [20] and neurodegenerative diseases [21], drug-induced liver injury [22], reproductive disorders [23], infections, cancer and ageing [24].

Ferroptosis is characterised by iron overload through NCOA4-dependent ferritinophagy, an insufficient antioxidant response leading to the peroxidation of firstly specific but ultimately most types of lipid particles, which induces necrotic-type cell death through a yet unclarified mechanism [25].

From a pharmacological perspective, specific inducers of ferroptosis (FINs) can function as highly efficient antitumor drugs that modulate iron homeostasis and the antioxidant system of the cancer cell. Canonical FINs erastin and RSL3 increase the labile iron pool through ferritinophagy and target the glutathione-dependent antioxidant system by inhibiting the glutathione biosynthesis precursor cystine or directly inhibiting the activity of the lipid peroxide scavenger glutathione peroxidase 4 (GPX4), respectively [26]. GPX4 modulation is the target of newly developed FINs, as FIN56 can specifically target GPX4 for degradation, while FINO2 directly inhibits GPX4 activity and its endoperoxide moiety oxidises cellular iron, thus initiating an in situ Fenton reaction and oxidative damage [26,27]. Although FINs are mostly still under development to reach clinical trials, many marketed anticancer drugs are associated with the induction of the ferroptotic pathway (for an extensive review, see [28]). Recent experiments showed that certain breast cancer lines can be potentiated towards iron overload-induced ferroptosis through the inhibition of NFS1 (cysteine desulfurase), the protein responsible for sulfur supply in the ISC biosynthetic pathway [29].

At the same time, hyperproliferative cancer cells are characterised by increased demand for iron, facilitating metastasis formation through iron overload and oxidative stress followed by glycolytic transformation and acidification of the cell’s microenvironment [30]. Moreover, it was shown that factors contributing to the epithelial–mesenchymal transition of cancer cells are directly related to ferroptosis sensitivity, which suggests susceptibility to ferroptosis-based therapies [28].

## 3. The Dual Role of ROS

The phrase “reactive oxygen species” (ROS) is commonly used to describe the highly reactive free radicals and molecules originating from molecular oxygen [31].

This so-called bi-radical state of oxygen explains its reactivity: one of its electrons can be paired with an external electron with an antiparallel spin, resulting in the production of the highly reactive superoxide radical (O_2_·^−^) [32,33]. Since the superoxide radical is weakly basic and highly soluble in water at physiological pH, cellular membranes are relatively impermeable to it [34]. However, O_2_·^−^ can be converted into membrane-permeable H_2_O_2_ by superoxide dismutase (SOD) or protonated to hydroperoxyl radicals (HOO·). Furthermore, O_2_·^−^ reacts with H_2_O_2_ through the Haber–Weiss reaction (Equation (1)) using iron catalysis, resulting in the formation of highly reactive and cytotoxic hydroxyl radicals (HO·) [1,33,34,35,36]. Furthermore, during the hydroperoxide and polyunsaturated fatty acid metabolism, other types of ROS, namely the peroxyl (ROO·) and alkoxyl (RO·) radicals, are formed as intermediates [36,37].

Singlet oxygen is also produced in many cellular reactions catalysed by a range of peroxidase enzymes, including myeloperoxidases, lactoperoxidases, chloroperoxidases and lipoxygenases. Since singlet oxygen is the lowest excited state of molecular oxygen, it is more reactive than (ground state) triplet oxygen. On the one hand, due to its relatively long lifetime in water solutions, singlet oxygen can be involved in the same reactions as triplet oxygen, but possibly with higher reactivity and efficiency [38,39]. On the other hand, singlet oxygen is a potent oxidant, promoting oxidative damage, so its levels must be kept under strict control, for example, by the consumption of foods rich in singlet oxygen quenchers, such as carotenoids [39,40]. These quenchers react with singlet oxygen and bring it back to its ground (triplet) state either by energy transfer or oxidative reactions [40].

### 3.1. Cellular Sources and Roles of ROS

**Figure 2:**
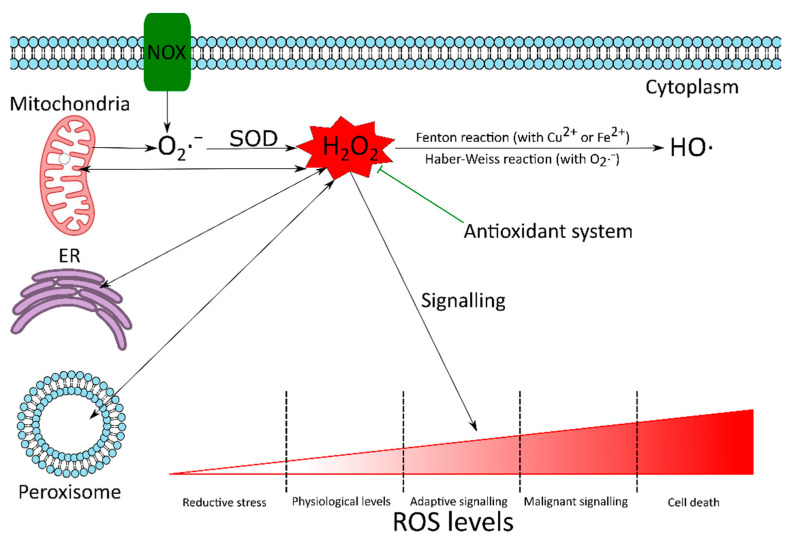
The dual role of ROS. There are many reactive oxygen species (ROS) sources in the cell. Although mitochondria are known to produce around 90% of the cellular ROS under physiological conditions, there are other notable sources too. These include NADPH oxidases (NOX), endoplasmic reticulum (ER) and peroxisomes. The superoxide radical (O_2_·^−^) produced in the cell is converted by superoxide dismutase (SOD) to hydrogen peroxide (H_2_O_2_). H_2_O_2_ can be converted to highly reactive and cytotoxic hydroxyl radical (HO·), via the Haber–Weiss (Equation (1)) or Fenton reactions (Equation (2)). Although ROS can cause oxidative damage to biomolecules, at strictly regulated levels they are required to maintain the redox homeostasis of the cell and are involved in adaptive signalling to overcome various stresses. If the antioxidant system fails to keep ROS under control, high ROS concentrations can initiate malignant signalling or cell death. Since H_2_O_2_ is relatively stable and can cross biological membranes, it is considered to be the most important redox signalling molecule.

ROS can be formed in many compartments of the cell (Figure 2). Among all compartments, mitochondria are the primary sources of ROS, since they are known to produce around 90% of the cellular ROS under physiological conditions [41]. This high ROS production rate is due to the involvement of mitochondria in oxidative phosphorylation, in which molecular oxygen is reduced to water. The mitochondrial electron transport chain may leak electrons, which results in the partial reduction of oxygen to O_2_·^−^. It has been reported that 0.2–2.0% of the molecular oxygen consumed by mitochondria is reduced to O_2_·^−^ [41,42]. Overall, 70–80% of O_2_·^−^ is generated in the Q cycle of complex III, but complex I is also considered to be a major source of mitochondrial ROS [43]. Glycerol 3-phosphate dehydrogenase, Q oxidoreductase, pyruvate dehydrogenase and 2-oxoglutarate dehydrogenase may also generate O_2_·^−^ [1,31,44]. Superoxide radicals are quickly transformed into H_2_O_2_ by SOD, which can be reduced to water by catalase or glutathione peroxidase, to avoid the formation of HO· [31,44,45].

There are various sources of ROS with cytoplasmic origin. The most significant are the members of the NADPH oxidase (NOX) family [44,46]. The NOX family has seven members: NOX1–5 and dual oxidase 1 and 2 (DUOX1 and 2). These membrane-bound enzymes transfer an electron from NADPH across a biological membrane to reduce oxygen to superoxide radical, which is then spontaneously or enzymatically dismutated to H_2_O_2_ (Figure 2) [1,31,44,47]. 

ROS are also produced in the peroxisomes and the endoplasmic reticulum (Figure 2). Peroxisomes are one of the primary sources of H_2_O_2_, which is produced as a by-product of the abundant peroxisomal oxidases, such as acyl-CoA oxidases, xanthine oxidase and D-amino acid oxidase [1,44,48]. In the presence of transition metal ions, H_2_O_2_ can be rapidly converted to highly reactive, cytotoxic HO· via the Fenton reaction (Equation (2)) [1,35,36,49]. The endoplasmic reticulum (ER) plays an important role in many cellular functions, such as protein synthesis and folding, posttranslational modifications, regulation of the secretory pathway and calcium storage [35,44,50]. Disturbances in the redox homeostasis or folding apparatus can lead to the accumulation of unfolded and misfolded proteins, which activate a survival mechanism, called unfolded protein response (UPR). UPR aims to help the cells to restore homeostasis, but it also induces ROS production, which may promote ER stress further, resulting in additional ROS production. If the balance is not restored and UPR is prolonged, it may trigger apoptosis [1,44,45,50].

Cells have developed various antioxidant and repair mechanisms to prevent the oxidative damage caused by ROS. If the systemic production of ROS exceeds the cell’s ability to restore the redox homeostasis, then the increasing ROS levels may have harmful effects on cellular structures and functions, resulting in so-called oxidative stress. Oxidative stress has been associated with the development of various degenerative processes, diseases, syndromes and other pathologies. Furthermore, oxidative stress has a role in tumour initiation, progression and resistance to therapy (Figure 2) [1,33,45,47,49].

However, at strictly regulated levels, ROS are involved in many physiological redox signalling pathways (Figure 2). These include chemotaxis, immune response, cytoskeletal remodelling, calcium homeostasis, growth, differentiation, cell cycle progression and cell death. Thus, the antioxidant system does not aim to eliminate ROS totally, but to keep them under control [1,31,44,48,49,51,52,53,54].

H_2_O_2_ is often considered to be the most important redox signalling molecule because it is relatively stable in vivo and can cross biological membranes. Nevertheless, the dipole moment of H_2_O_2_ is higher than that of water, which severely limits its passive diffusion across biological membranes. The efficient transport of this molecule requires specific channel proteins, namely the aquaporins [35,48]. These give the cells the capability to spatially separate H_2_O_2_-producing and -consuming reactions and form intracellular H_2_O_2_ gradients to regulate the response of redox-sensitive systems finely [48].

In addition to normal physiological processes, ROS signalling may be involved in malicious processes, depending on cell type, environment and ROS source [44]. The involvement of ROS in malignant signalling also highlights the need for the elements of the antioxidant system to act orchestrated. For example, SOD increases the levels of H_2_O_2_. Without sufficient activity of enzymes that catalyse the conversion of H_2_O_2_, increased H_2_O_2_-dependent signalling may occur, resulting in various pathologies [55].

### 3.2. ROS in Cancer

Elevated ROS production can be observed in cancer cells compared to normal cells [1,33,35,36,45]. Multiple factors can have a role in increased ROS generation, such as enhanced metabolic activity, mitochondrial dysfunction, peroxisome activity, increased cellular receptor signalling, oncogene activity, and increased activity of ROS producing enzymes such as oxidases, cyclooxygenases, and lipoxygenases [35,56]. ROS in cancer cells may amplify the tumorigenic behaviour and facilitate the development of additional mutations promoting metastasis [33].

The oncogene signals that boost ROS production in cancer cells also reprogram the antioxidant system to withstand the constant oxidative stress and keep ROS production at nontoxic levels [46,56]. Accordingly, increased catalase levels were reported in breast cancer tissues, malignant mesothelioma and colorectal carcinoma. Furthermore, in addition to peroxisomal catalase, malignant cells may acquire membrane-associated catalase to increase their survival under oxidative stress. At the same time, several other studies found the downregulation of catalase [35,46,56].

ROS also increase the expression of genes under the control of nuclear factor erythroid 2 (Nrf2). These genes encode enzymes that take part in the biosynthesis, utilization and regeneration of glutathione, thioredoxin and NADPH. Furthermore, the expression of other antioxidant enzymes under the control of activator protein 1 (AP-1), nuclear factor κB (NF-κB), hypoxia-inducible transcription factor 1α (HIF-1α) and p53 is also increased [31,46,56].

Autophagy is also induced by ROS. Autophagy enhances cancer cell survival by increasing stress tolerance and supplying nutrients to meet the high metabolic and energetic demands of proliferating cancer cells by recycling biomolecules [46,57].

The induction of antioxidant systems and autophagy provides an interconnected and finely adjustable mechanism for cancer cells to survive and maintain the oxidative stress required for tumour development and progression [46,58]. Disturbing the precise function of this adaptive mechanism allows us to elevate the oxidative pressure in cancer cells and thus selectively kill them. Such strategies have been demonstrated and used effectively previously [1,35,36,56].

However, it must be noted in general that the responses to ROS are highly complex and dependent on multiple factors, including the origin, the environment and stage of the tumours, and the types, levels, localisation and persistence of ROS (for an extensive review, see [55]).

### 3.3. ROS in Ischemia-Reperfusion Injury

Ischemia-reperfusion injury (IRI) occurs when the blood flow to an organ is interrupted (ischemia) and then re-established (reperfusion). IRI has a central role in the pathology of major cardiovascular diseases, such as stroke and myocardial infarction, and in organ injuries after their transplantation [59,60,61,62]. 

IRI is mediated by several factors, including ROS, produced in great amounts after reperfusion [59,60]. However, the main source of ROS during this oxidative burst is controversial, and it probably depends on tissue type. For example, mitochondria seem to be the primary ROS sources in the metabolically highly active brain and heart, while xanthine oxidase in the intestine. Furthermore, the physiological status of the tissue may also influence the relative contribution of enzymes to ROS production; for example, cytokines may up-regulate the expression of certain ROS-producing enzymes in inflamed tissues [62].

The mechanisms involved in the pathogenesis of IRI are multifactorial, complex and highly integrated [60]. During reperfusion, increased ROS production and mitochondrial calcium overload were observed. These are believed to play a critical role in the opening of mitochondrial permeability transition pore (mPTP), which is a crucial element of reperfusion damage [59,60,63]. mPTP is a non-selective channel across the inner and outer mitochondrial membranes, which allows the exchange of solutes between the mitochondrial matrix and the cytoplasm up to 1500 Da. The opening of mPTP is associated with the drastic and sustained depolarization of mitochondria and a burst of ROS production. The increased O_2_·^−^ production due to mPTP opening may lead to further mPTP opening in adjacent mitochondria and eventually to cell death [59,62,64].

ROS may also act as signalling messengers to protect the cells and tissues against lethal oxidative stress induced by ischemia and subsequent reperfusion. During ischemic preconditioning (IPC), short, non-lethal cycles of ischemia and reperfusion are applied [59,60,65,66,67]. The moderate amounts of ROS produced during IPC activate signal transduction pathways by posttranslational modification of redox-sensitive proteins. The inhibition of mPTP opening is considered to be the final step of these pathways [59]. IPC also triggers further adaptive changes in the cells and tissues, including better electrolyte and acid-base homeostasis, lower ROS production, reduced inflammatory responses and improved microcirculatory perfusion [66]. All these changes protect the preconditioned tissues against severe IRI [59,60,65,66,67].

### 3.4. ROS in the Defence against Pathogens

High concentrations of ROS are not only harmful to the cell producing them but also to invading pathogens. Phagocytes such as macrophages, neutrophils, and dendritic cells generate large amounts of ROS in the phagolysosomes to kill microbes during the earliest stages of pathogen interactions [33,47,68,69,70]. 

The multi-subunit NOX2 mediates the phagocyte oxidative burst. After its assembly on the phagolysosomal membrane, NOX2 pumps electrons into the compartment to reduce molecular oxygen to O_2_·^−^, which is then dismutated to membrane-permeable H_2_O_2_. Furthermore, highly cytotoxic HO· is also formed via the Haber–Weiss reaction. In addition, the myeloperoxidase, found in the granules of neutrophils, catalyses the formation of further oxidants, such as hypochlorous acid, hypothiocyanous acid and hypobromous acid from H_2_O_2_ [33,47,71]. These compounds kill the engulfed microbes by oxidizing their biomolecules, including their DNA [68,70]. 

ROS produced by NOX2 also play a role in the formation of neutrophil extracellular traps (NETs) by activating granular proteases. NETs are extracellular structures composed of DNA and granule proteins, such as histones, neutrophil elastase, myeloperoxidase and cathepsin G. In these traps, extracellular pathogens are caught and killed [47,69,72].

Many loss-of-function alleles have been described for NOX2 NADPH oxidase subunits in humans, making NOX2 unable to produce ROS. The inability of phagocytes to produce ROS renders the innate immune system inefficient and results in chronic granulomatous disease. Patients with this life-threatening primary immunodeficiency suffer from recurrent bacterial and fungal infections and granuloma formation [33,47,68,69,73]. ROS are not only required in defence against pathogens, but to limit inflammation and immune response. Accordingly, the lack of NOX2-produced ROS is also associated with severe autoimmune diseases, such as autoimmune arthritis [73,74,75].

## 4. Ascorbate: The Molecule of Clichés

Ascorbate is considered to be the molecule of clichés. What is the most common cliché? That it is an excellent antioxidant, it scavenges the different ROS, and it protects the cell against cell death? Let’s see behind the curtain of this cliché!

### 4.1. The Excellent Antioxidant

According to the pH, the molecule can exist in the protonated ascorbic acid (AH_2_) form, that by the loss of a proton forms the ascorbate anion (AH^−^) at higher pH (pKa,1 = 4.1; pKa,2 = 11.8) [76]. Hence, the ascorbate monoanion is the dominant form at physiological pH. Both forms can donate an electron to strong oxidizing agents such as superoxide (O_2_·^−^), hydroperoxyl radical (HOO·) or hydroxyl radical (HO·) and form the ascorbate radical (A·^−^). Since the unpaired electron of the ascorbate radical is delocalised in the π-systems of orbitals, A· ^−^ is relatively unreactive [77]. It decays relatively slowly compared to the abovementioned highly reactive radicals HOO· and HO·. At physiological pH, the main form of decay is the disproportionation of A·^−^. The dimer formation of A·^−^ during the disproportionation gives further stability to A·^−^. The products of the disproportionation are the harmless AH^−^ and dehydroascorbate (DHA); furthermore, DHA can be recycled quite easily [35,78]. The observation that AH^−^ is an excellent chain breaking antioxidant is based on these chemical features.

The cell organelles where ROS generation meets with cell death and ascorbate are the mitochondria. These organelles are responsible for the generation of the vast majority of ROS in human cells [43] and also play important roles in different (ROS-driven) cell deaths [79]. In this way, they gave excellent field to study the antioxidant role of ascorbate.

It was found quite early that the intra-mitochondrial ascorbate level can play an important role in the mitigation of ROS-driven cell dell death. The elevated level of ROS can induce the collapse of the mitochondrial membrane potential, which leads to apoptosis. Several studies showed that the incubation of cells with DHA before direct hydrogen peroxide (H_2_O_2_) treatment or treatments which lead to elevated H_2_O_2_ generation such as hypoxia and reperfusion or FAS-induced apoptosis of monocytes could mitigate the cell death. In further detail, DHA preincubation could reduce the ROS levels in a dose-dependent manner, along with the denaturation and mitochondrial release of cytochrome c [80], and the mitochondrial membrane potential was also partially conserved [81,82]. As a result, the activation of caspase-9 and caspase-3 was decreased with a consequent inhibition of apoptosis.

The vast majority of superoxide produced in the cytosol and mitochondria is converted to H_2_O_2_ by the Cu- and Zn-containing SOD1. Different non-lethal abnormalities were found in SOD1 knock out mice, such as female infertility [83], vascular dysfunction [84], accelerated skeletal muscle atrophy [85], retinal dysfunction [86], a malfunction of lipid metabolism [87], and anaemia [88]. Their lifespan decreased by 30%, and hepatic nodular hyperplasia or hepatocellular carcinoma was developed in 70% of the animals in later life [89]. At the same time, mouse embryonic fibroblasts from SOD1 knock out mice died under normoxic culture (20% oxygen). The level of ROS and lipid peroxidation was increased. The immediate cell death could be prevented by hypoxic culture (2% oxygen); however, it could not recover the proliferative ability of the SOD1 knock out cells [90]. Ascorbate supplementation could ameliorate the oxidative stress due to the depletion of SOD1 or SOD2 and rescues the fatal phenotype [91,92]. The double SOD1 and aldehyde reductase (AKR1a, deficient in ascorbic acid biosynthesis) mice served as evidence that ascorbic acid plays a crucial role in the elimination of superoxide. AH_2_ was essential for the double knock out mice to breed, and all of them died within two weeks after the withdrawal of AH_2_ supplementation [93]. After the termination of AH_2_ supplementation, their blood plasma levels started to decline (the half-life of AH_2_ is approximately one day), accompanied by the initiation of pathological damage. After the examination of the principal organs, it could be assessed that the central nervous systems and kidneys showed no evident damage after the termination of AH_2_ supplementation. The liver and heart showed only moderate pathological changes. However, the AH_2_ depletion caused hyperaemic oedema of the lungs with invaded leukocytes, indicating that pulmonary dysfunction was the direct cause of death [93]. The serious oxidative stress of the pulmonary tissue was also reinforced by the elevated 8-OHdG level of pulmonary cells after AH_2_ withdrawal. The severe oxidative damage of the lung compared to the peripheral tissues could be explained by the hyperoxic conditions of the lung. Although, on the base of the rate constant (2.7 × 10^5^ M^−^^1^s^−^^1^), AH_2_ is not a very effective superoxide scavenger, its high tissue concentration (0.5–10 mM) makes it an important player in the fight against excess superoxide generation. This cooperation of SOD and AH_2_ in the ROS-antioxidant battle was also underlined by the observation that the plasma level of AH_2_ in mice deficient in CuZn-SOD was decreased by 25% [94].

### 4.2. How can Ascorbic Acid Give Solutions to Everyday Problems?

Cardiac fibroblasts play a crucial role in the coordination of tissue repair after ischemia-reperfusion (IR)-caused myocardial damage. Ascorbic acid could increase the viability of cardiac fibroblasts exposed to simulated IR (sIR) in a concentration-dependent manner [95]. Beyond the free radical scavenger antioxidant ascorbic acid, the free iron chelator deferoxamine and the reduced glutathione (GSH) precursor N-acetylcysteine showed similar effects [95]. The beneficial effect of their combination far exceeded the effect of each compound separately. The cocktail of ascorbic acid, deferoxamine and N-acetylcysteine could elevate the viability of cardiac fibroblasts exposed to sIR in a lower concentration than those of each antioxidant which, separately, had a cytoprotective effect only at higher concentrations. The cytoprotective effect of the cocktail was associated with decreased intracellular ROS production and apoptotic cell death induced by sIR. It could also activate the pro-survival kinases ERK1/2 and Akt, but inhibited the pro-apoptotic p38 and JNK kinases. The protection of cardiac fibroblasts due to the cocktail accompanied by wound repair induced by sIR by restoring serum-induced migration, TGF-β1-mediated differentiation of cardiac fibroblasts into cardiac myofibroblast and angiotensin II-induced pro-collagen I synthesis [95].

In another study, the early death of engrafted cells during the cell therapy of myocardial infarction could be mitigated by an ascorbic acid-containing cocktail. The cocktail of eicosapentaenoic acid, docosahexaenoic acid and ascorbic acid could mitigate the cell death of embryonic stem cell-derived cardiac lineage cells in the harsh oxidative stress environment. In this way, the pre-treatment and the injection of the embryonic stem cell-derived cardiac lineage cells into the ischemic myocardium of a rat model of myocardial infarction significantly could reduce the fibrosis compared to the vehicle group [96].

Similarly, the potent anti-promyelocytic leukaemia drug, arsenic trioxide (As_2_O_3_), induced the depletion of total antioxidant capacity, and the mitochondrial transmembrane potential of cardiomyocytes could be mitigated by the co-treatment of cardiomyocytes with ascorbic acid and α-tocopherol. This co-treatment resulted in a significant reversal of oxidative stress and the alteration of the antioxidant defence through the activation of Nrf2 and Bcl2, which led to increased cell survival and prevented apoptosis [97]. Another anticancer agent, doxorubicin also induces the elevation of ROS in cardiomyocytes in a time-dependent manner that follows the activation of stress-induced proteins such as p53, p38 and JNK MAPKs, leading to an increase in autophagy and apoptosis. A concentration of as low as 25 μM of ascorbic acid could alleviate the increase in ROS due to doxorubicin treatment accompanied by the attenuation of the activation of signalling pathways, leading to the prevention of apoptosis and preservation of cell viability [98]. This way, ascorbic acid may potentially be used as an antioxidant adjuvant therapy to avoid the doxorubicin-induced cardiomyopathy [98].

Necessarily, the cardioprotective effect is just one example to demonstrate the general ROS scavenging and consequent cytoprotective (anti-cell death) effect of ascorbic acid.

### 4.3. Outside of the Cliché: The Pro-Oxidant Role of Ascorbate

Although the pro-oxidant role of ascorbate is outside of the clichés associated with this molecule, it is as important as the antioxidant role, and this makes ascorbate a real “dual function molecule” that plays an important role in “cell fate decision”.

Despite its well-known antioxidant role, it can cause oxidative damages through the Fenton reaction (Equation (2)) [35]. The occurrence of this pro-oxidant effect requires the presence of transition metal ions. Ascorbic acid reacts overwhelmingly with the oxidised forms of iron and copper [76]. Earlier, three different mechanisms have been proposed to describe the oxidation of ascorbic acid by iron and copper [76]. They share a similar initial step: the oxidised form of the metals react with oxygen, producing A·^−^ or DHA and a reduced form of oxygen (HOO·, O_2_·^−^ or H_2_O_2_). This transfer of one or two electrons between the ascorbic acid and the oxygen can occur through the metal ion bridge. It was described recently that as low as micromolar concentrations of iron(III) and copper(II) behaved as important sinks for ascorbic acid (both AH_2_ and AH^−^). It was found that their role is more important than those of ROS. In this way it was proposed that the iron and copper reactions are catalytic rather than redox ones [76].

**Figure 3:**
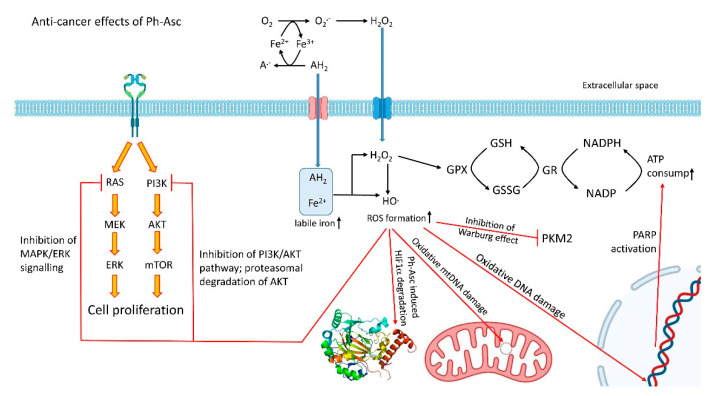
The anti-cancer effects of Ph-Asc. Pharmacologic ascorbate (Ph-Asc) in the presence of transition metals such as iron (Fe) induces the generation of H_2_O_2_ and the most powerful known oxidizing agent, hydroxyl radical. In this way, Ph-Asc treatment induces extended oxidative DNA damage that initiates PARP1-dependent repair process which lead to significant NAD^+^ and consequent ATP consumption. mtDNA proved to be more susceptible due to its less efficient repair system. Ph-Asc also induces the PHD2-independent downregulation of HIF-1α. Ph-Asc could inhibit the phosphorylation of PKM2 that halted the nuclear translocation of PKM2, resulting in the impairment of the Warburg effect in cancer cells and xenografts. Finally, Ph-Asc treatment could significantly inhibit the activity of both MAPK/ERK and PI3K/AKT signalling. All the observed cancer cytotoxicity could be reversed by intracellular or extracellular catalase supplementation.

In the forthcoming steps, H_2_O_2_—in the presence of millimolar concentrations of ascorbate (which is called pharmacological ascorbate; Ph-Asc)—can readily react with further transition metal ions in the Fenton reaction to form the highly reactive, cytotoxic hydroxyl radical [35]. In this way, ascorbate contributes to the continuous generation of ROS (Figure 3).

The antioxidant role of ascorbate contributes to the mitigation of oxidative stress and oxidative stress-driven cell death (see the chapter above). The pro-oxidant role of ascorbate ties the molecule to the generation of excess ROS that leads to cell death. Necessarily, different cells show different sensitivity to oxidative stress.

It is generally accepted that cancer cells can be characterised by persistent oxidative stress. It seems that cancer cells keep a fine redox balance that ensures their proliferation but avoids their cell death. Therefore, agents that induce the generation of increased ROS levels are frequently used as cytotoxins in cancer patients [99].

It is not accidental that the anti-cancer effect was attributed to pharmacological ascorbate as early as 40 years ago [100]. Although several important discoveries have been made with regard to its anti-cancer action, the exact mechanism has not been elucidated to this day.

Since all of its anti-cancer effects could be suspended by enzymatic and non-enzymatic antioxidant treatment, the ROS generated by Ph-Asc treatment seem to have a central role [35]. Ph-Asc can exert this beneficial effect by fighting the cancer cells at multiple points:

#### 4.3.1. Rupture of Bioenergetics

For a long time, the bioenergetic collapse of cancer cells due to Ph-Asc treatment has been considered to be the major factor behind its anti-cancer effect. Indeed, almost an immediate depletion of intracellular NAD^+^ and ATP can be observed after Ph-Asc treatment [101]. The decrease in NAD^+^ and ATP stores could be prevented by extracellular catalase, confirming the contribution of H_2_O_2_ to the disruption of cellular bioenergetics upon Ph-Asc treatment. The analysis of the time courses of Ph-Asc treatment led to the establishment of the idea that the changes in cellular bioenergetics following the treatment were the result of increased demand due to DNA damage and not the changes in the production rate of ATP [101].

#### 4.3.2. DNA Damage

The generation of the most powerful known oxidizing agent, hydroxyl radical, is an important consequence of Ph-Asc treatment (see above). Since DNA is considered to be the primary target of the formed hydroxyl radical [101], DNA double-strand breaks could be observed in pharmacological ascorbate-treated neuroblastoma, pancreatic, and ovarian cancer cells [102,103]. Similarly to the disruption of cellular bioenergetics, these DNA double-strand breaks could be prevented by extracellular catalase [104], reinforcing the role of H_2_O_2_. As was expected—because of its less efficient repair systems—mitochondrial DNA was more susceptible to oxidative damage due to Ph-Asc treatment than the nuclear DNA. According to the expectations, the Ph-Asc treatment-induced extended DNA damage initiated the PARP1 dependent repair process that led to significant NAD^+^ and consequent ATP consumption (Figure 3). This phenomenon could be observed both in vitro in pancreatic cancer cells and in vivo in tumour xenografts from mice treated with infusions of Ph-Asc [101].

Both NAD^+^ and ATP levels could be preserved by the deletion or by the chemical inhibition of PARP-1, while the toxicity of Ph-Asc on cancer cells remained. According to these observations, Ph Asc-induced disruption of bioenergetics seems to not be the primary factor for the toxicity of Ph Asc [101].

#### 4.3.3. Accelerated Degradation of HIF-1α, Decreased Adaptation to Hypoxia

The loss of contact inhibition, the outgrowth of vasculature, and increased oxidative phosphorylation result in a locally hypoxic tumour environment. Hypoxia-inducible transcription factors (HIF-1, 2, and 3) play a crucial role in the management of hypoxia. Briefly, HIF heterodimer consists of an oxygen-dependent α-subunit and a constitutively expressed β-subunit. During hypoxia, HIF-1α enters the nucleus, forms a dimer with HIF-1β, and the dimer binds to DNA through the hypoxia response elements (HREs). HREs are responsible for the transcription of genes involved in metabolism, angiogenic signalling including vascular endothelial growth factor (VEGF), vasomotor regulation, matrix and barrier function, growth, apoptosis [105]. Exposure to hypoxia—through the induction of HIF-1α—stimulates the production of VEGF that is a potent angiogenic stimulant [106]. On the one hand, angiogenesis supports tumour growth; on the other hand, it may also provide a route for cells to metastasise [107]. Under normoxic conditions, two proline and one asparagine residues of HIF-1 are hydroxylated, inhibiting HIF-1. Furthermore, hydroxylation of prolyl residues—mainly by PHD2—increases its affinity for the von Hippel–Lindau ubiquitin ligase complex, leading to the proteolysis of HIF-1α [108,109]. Not surprisingly, the malignant features of tumours, which expressed high HIF-1α levels were higher, and the selective inhibition of HIF-1α with natural degradation pathways that bypass the common PHD2 pathway could suppress these features [110].

Wilkes et al. [111] found that Ph-Asc-induced cytotoxicity correlated with the increased degradation of HIF-1α (Figure 3). The effects on HIF-1α and cell cytotoxicity could be reversed by intracellular or extracellular catalase supplementation. Downstream from HIF-1α, VEGF could also be inhibited in a similar dose-dependent manner. Although the original observations were obtained on MIA PaCa-2 cells, the slowed tumour growth and inhibition of VEGF production could be translated to in vivo xenograft mouse model as well [111]. Interestingly, the Ph-Asc-induced HIF-1α downregulation was independent of PHD2 [111]. Since HIF-1α is a major transcription factor in the development of metastases, another benefit of the Ph-Asc treatment could be the inhibition of mechanisms that drive metastases.

#### 4.3.4. Impairment of Hybrid and Warburg Metabolism

The hybrid metabolism observed in several cancer types enables them to switch between glycolytic and oxidative pathways that contribute adaptations to the various tumour microenvironments. In this way, the Warburg effect and the hybrid metabolism can serve as potential therapeutic targets [112]. Pyruvate kinase M2 (PKM2) plays a central role in the regulation of the Warburg effect. PKM2 is overexpressed in cancer cells [113], and furthermore its monomer form can translocate into the nucleus and upregulates the expression of c-Myc and cyclin D1. The expression of c-Myc is upregulated in many types of cancers, and its overexpression results in the enhanced expression of glucose transporter GLUT1 and elevated activity of the glycolytic enzymes (HK2, PFK, enolase1) and LDHA with the overproduction of lactic acid [114]. It should also be emphasised that c-Myc is able to upregulate PKM2, generating a positive feedback loop [115]. Recently, it was shown that pharmacologic ascorbate could induce RAS detachment from the cell membrane inhibiting ERK 1/2 and PKM2 phosphorylation. As a consequence of this activity, strong downregulation of the glucose transporter (GLUT1) and PKM dependent protein expression could be observed, resulting in the impairment of the Warburg effect in KRAS mutant cells and xenografts (Figure 3) [116]. Interestingly, Ph-Asc treatment did not cause any change in the mRNA expression of PKM2, but it inhibited the phosphorylation of PKM2 that halted the nuclear translocation of PKM2. These observations suggest that Ph-Asc exerted its anti-cancer effect at least partly through the inhibition of the Warburg effect.

#### 4.3.5. The Halt of MAPK/ERK and PI3K/AKT Signalling

Ph-Asc treatment could significantly inhibit the activity of both MAPK/ERK and PI3K/AKT signalling in a dose-dependent manner in thyroid cancer cells. Furthermore, it was found that Ph-Asc promoted AKT proteolysis by the ubiquitin-proteasome degradation pathway (Figure 3) [117]. All the observed effects of Ph-Asc could be reversed by N-acetylcysteine treatment, suggesting the role of elevated ROS in the background. As a continuation of the previous work, it was demonstrated that Ph-Asc could sensitise BRAF mutant thyroid cancer cells to vemurafenib [118]. Ph-Asc could relieve the feedback activation of MAPK/ERK as well as PI3K/AKT pathways induced by vemurafenib (Figure 3). As a result, the co-therapy of vemurafenib and Ph-Asc suppressed the malignant progression of thyroid cancer [118].

## 5. Conclusions

Although the three main subjects of our review can be seen to be different at first glance, they show several common features. First, the control of their level is critical. It is not accidental that the uptake, transport and storage of both iron and ascorbic acid are strictly controlled. The appropriate iron support is critical at both the cellular and the whole-body level. Iron is a critical component of numerous proteins, and iron deficiency is accompanied by reduced oxygen-carrying capacity of the blood. At the same time, the uncontrolled uptake and level of free iron leads to the production of potentially toxic ROS. The cellular and whole-body level of ascorbate is also strictly controlled by its uptake and recycling. It gives special protection in low or moderate concentrations against ROS generated in different processes, such as IR or drug-induced toxicity. At the same time, ascorbate in high doses in the presence of transition metals—such as iron—induces the production of the aggressive oxidizing agent H_2_O_2_ and hydroxyl radical, which leads to oxidative stress and oxidative stress-induced cell death. The dual role of all these entities can be further boosted by the observation that cancer cells are more sensitive to oxidative stress than normal cells. In this way, the generated excess ROS (due to high dose ascorbate treatment) can be life-savers via the killing of cancer cells or different pathogens.

In the future, the elucidation of the details and the links between the iron, ROS, oxidatively damaged phospholipids driven ferroptosis and high-dose ascorbate-induced cancer-selective cell death mechanisms may give us a tool to develop and apply efficient cancer therapies. Since redox signalling pathways, enzymatic and non-enzymatic antioxidant systems are involved in the regulation of ferroptosis via the control of the cellular redox status, their more detailed knowledge shall be critical in this development. The observations that ferroptosis is commonly suppressed in cancer cells and that cancer cells which are resistant to common chemotherapeutic drugs seem to be highly susceptible to ferroptosis inducers [119] underlie the great potential of the modulation of ferroptosis via the redox reprogramming in cancer treatment.

## Data Availability

Not applicable.
